# Multivariate genetic architecture of age-related eye disease

**DOI:** 10.1371/journal.pone.0349199

**Published:** 2026-05-14

**Authors:** Luqi Gao, Qihao Wang, Chao Zhu

**Affiliations:** The Second Hospital of Jilin University, Changchun, China; University of Melbourne, AUSTRALIA

## Abstract

At present, the genetic architecture underlying traits linked to Age-related eye disease (ARED) remains largely unexplored. We utilized Genomic Structural Equation Modeling (Genomic-SEM) and various Post-processing analysis of Genome-Wide Association Studies (GWAS) to identify statistically prioritized candidate single nucleotide polymorphisms (SNPs) associated with independent ARED variants. A total of 11 genome-wide significant loci were identified in the study. By applying diverse transcriptome-wide association approaches, we analyzed tissue-, cell layer-, and genomic element-associated gene signals reflecting age-related ocular vulnerabilities, alongside their functional annotations in relation to ARED. Through conducting a GWAS on a phenotype not directly measured, our research presents the first comprehensive genetic landscape of ARED.

## Introduction

Vision constitutes a fundamental aspect of human health [1]. With the global population aging, the prevalence of eye diseases leading to vision impairment and blindness is on the rise. According to estimates from the Global Burden of Disease (GBD) study, approximately 295 million individuals worldwide experienced moderate to severe vision impairment (VI) in 2020, with 43.3 million experiencing blindness [2]. Age-related eye disease (ARED), including senile cataract (SC), age-related macular degeneration (AMD), glaucoma(Gla), and diabetic retinopathy (DR) are becoming increasingly significant public health concerns [3]. ARED involves complex and interconnected mechanisms, characterized by physiological functional decline, diminished cellular regenerative capacity, and profound influences from genetic, environmental, and lifestyle factors [4,5]. As global aging accelerates, the rapid rise in ARED incidence poses major challenges in both medical and socioeconomic domains. Medically, the growing prevalence of these chronic conditions strains healthcare capacities due to the continuous need for advanced diagnostics, specialized care, and long-term therapeutic interventions. Socioeconomically, the impact is equally profound; the Lancet Global Health Commission estimates that vision impairment poses an enormous global financial burden, with the annual global cost of productivity losses reaching approximately US$ 411 billion [6]. Furthermore, severe vision loss significantly reduces patient independence, necessitating extensive informal caregiving and compounding the financial and emotional toll on families and broader health systems. Despite recent advances in senile cataract research, the specific genetic and biological underpinnings of ARED remain incompletely understood [7]. While aging has been identified as a key driver of ARED, existing findings still fail to fully explain interindividual variability in disease progression and susceptibility [8].

To address these challenges, this study aims to integrate multiple genetic analysis tools and high-correlation exploration methods to investigate potential molecular mechanisms and expand associations with diverse disease pathways. Specifically, we focus on identifying genomic loci and chromosomal regions linked to ARED, thereby uncovering potential therapeutic targets. Our work not only advances the understanding of ARED but also provides theoretical and practical support for interventions targeting aging and vision loss.To address the current lack of precise measurements for ARED mechanisms, we designed a Genome-Wide Association Studies (GWAS) targeting latent, unmeasured ARED phenotypes. Utilizing Genomic Structural Equation Modeling (Genomic-SEM) [9], we applied this approach to published GWAS summary statistics of ARED-related traits. These statistics enabled us to estimate single nucleotide polymorphism (SNP) associations with latent ARED phenotypes, thereby establishing a GWAS for ARED.

In this study, the term ARED is utilized as a broad epidemiological classification, distinct from the specific conditions targeted in the ARED Studies (AREDS/AREDS2) clinical trials. We specifically focused on four major ARED — SC, AMD, Gla, and DR — which collectively constitute the leading causes of moderate-to-severe vision impairment and blindness among the elderly worldwide [6]. Biologically, despite distinct primary clinical triggers, their disease trajectories converge on shared degenerative mechanisms—namely, cumulative oxidative stress, cellular senescence, and chronic neuroinflammation within the ocular microenvironment [10]. The inclusion of these four specific traits was determined by strict criteria: the availability of high-quality, large-scale GWAS summary statistics, their profound global public health burden, and their demonstration of sufficient univariate heritability and genetic covariance required to support robust multivariate genetic analyses via Genomic-SEM. Furthermore, while DR is primarily initiated by systemic hyperglycemia, its progression involves oxidative stress, neuroinflammation, and retinal vascular senescence — mechanisms deeply intertwined with the age-related ocular degenerations seen in AMD and Gla. Therefore, we hypothesized that these four conditions share a downstream, genetically driven vulnerability within the ocular microenvironment.

Building on systems biology methodologies, we defined the portion of ARED genetic variation unexplained by known biomarkers as potential genetic markers and conducted multiple GWAS-related analyses. While this approach is not exhaustive in capturing the intricate interplay between age-related pathways and multifactorial interactions—given that ARED are complex processes driven by genetic, environmental, and stochastic factors—it mitigates confounding effects from conventional ARED biomarkers, allowing analysis of otherwise intractable data [11]. In this study, the latent ARED phenotype extracted via Genomic-SEM is defined as a composite biological construct. It captures the intersection where systemic aging vulnerabilities drive shared structural and functional degeneration across the ocular microenvironment. From a translational perspective, we developed a simplified ARED risk factor atlas to enable non-biostatisticians to directly apply these insights for patient-specific prevention and intervention strategies. Our study aims to bridge the gap between genomic statistics, foundational research, and clinical implementation.

## Materials and methods

Our input GWAS datasets were derived from four studies focusing on ARED: SC, AMD, Gla, and DR. All datasets obtained ethical approval from their respective institutional review boards, with participants providing informed consent and data undergoing rigorous quality control. Specifically, the GWAS data for DR (n = 432,209) were derived from Verma et al. [12], while SC (n = 493,421) and AMD (n = 474,181) datasets were obtained from the FinnGen consortium (https://www.finngen.fi/en) [13]; Gla data (n = 462,933) were sourced from the IEU OpenGWAS project (https://gwas.mrcieu.ac.uk) [14]. (The specific cohort metadata, including the precise OpenGWAS dataset identifiers used for each trait, are comprehensively detailed in S1 Table.)

### Quality control for univariate GWAS

Low-quality sample exclusion: Samples with missing rates exceeding 5% were excluded. Special handling was applied to the MHC (Major Histocompatibility Complex) region on chromosome 6 (genomic coordinates: ~ 25,000,000–35,000,000 bp) due to its genetic diversity, structural complexity, and high polymorphism of immune-related genes [15,16]. For summary statistics preparation, default parameters were employed. All autosomal SNPs from four input GWAS datasets of ARED underwent recommended quality control filtering against the 1000 Genomes Phase 3 EUR reference panel [17]. The following filters were applied: (1) Removal of SNPs with MAF < 0.01 (low-frequency variants are prone to genotyping errors and exhibit higher standard errors in Linkage Disequilibrium Score Regression (LDSC) [18]; (2) Exclusion of SNPs with zero effect size estimates (to prevent matrix reactivity issues, essential for Genomic-SEM); (3) Removal of SNPs incompatible with the reference panel; (4) Exclusion of allele-mismatched SNPs.

### Sample overlap in univariate GWAS

A major methodological advantage of employing Genomic-SEM is its robustness to potential or unknown sample overlap among the input univariate GWAS datasets. Genomic-SEM utilizes multivariable LDSC to estimate the empirical genetic covariance matrix. Within this framework, the cross-trait LDSC intercept explicitly captures and adjusts for inflation and phenotypic correlations arising from shared participants or cryptic relatedness across the diverse genomic repositories. Consequently, the model inherently corrects for any latent sample overlap without requiring the manual identification or exclusion of overlapping individuals, thereby ensuring unbiased genetic correlation and structural parameter estimates. Because this study relies exclusively on summary-level statistics, individual-level phenotypic overlap (comorbidity) cannot be directly ascertained. However, the multivariable LDSC framework inherently quantifies and stringently corrects for any unknown sample overlap and cryptic relatedness across the input ARED cohorts through the calculation of bivariate cross-trait intercepts, ensuring that the latent factor structure is not statistically confounded by shared individuals.

### Genomic-SEM

**Fig 1:**
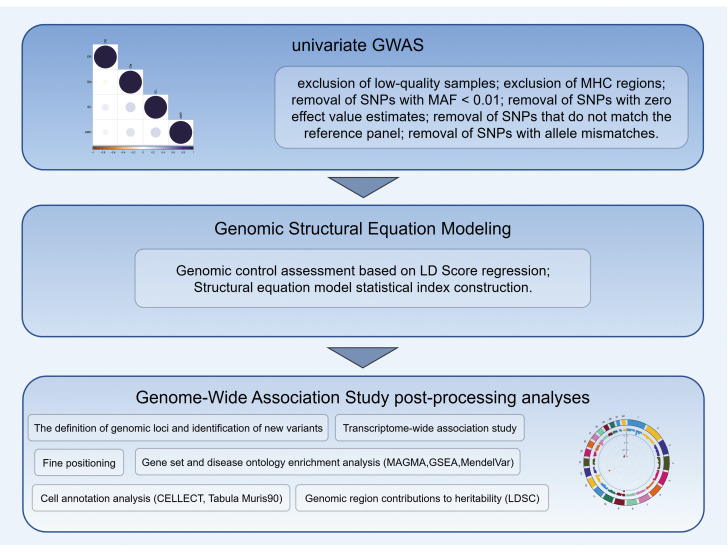
Flowchart detailing the study design and analytical pipeline. The workflow encompasses the data preprocessing of single-input GWAS for four age-related eye diseases, multivariable Genomic Structural Equation Modeling (Genomic-SEM) to extract the latent ARED factor, and comprehensive post-processing analyses. SC: Senile cataract, Gla: Glaucoma; AMD: Age-related macular degeneration; DR: Diabetic retinopathy; GWAS: Genome-Wide Association Study; MAGMA: Multi-marker Analysis of GenoMic Annotation; TWAS: Transcriptome-Wide Association Study. (By Figdraw.).

We employed Genomic-SEM implemented in the GenomicSEM R package (v.0.0.5) to conduct GWAS on ARED, including SC, AMD, Gla, and DR. This analysis aimed to investigate the broad genetic susceptibility underlying these AREDs (Fig 1). Genomic-SEM is a recently developed methodological framework [9] that enables the examination of multiple latent multivariate models, thereby facilitating exploration of the underlying architecture of traits of interest. (Refer to Table 1 for detailed criteria.)

**Table 1:** Detailed parameters of each GWAS data in the structural equation modeling.

Phenotype	NSNPs	h2 (se)	λGC	Mean ChiSquare	Intercept (se)	Ratio (se)
SC	1160411	0.025 (0.002)	1.2549	1.3473	1.1027 (0.0121)	0.2956 (0.0348)
Gla	1001811	0.0124 (0.0015)	1.0966	1.1438	1.0232 (0.0099)	0.1611 (0.069)
AMD	1160320	0.0171 (0.0054)	1.1575	1.2462	1.0895 (0.0189)	0.3634 (0.0768)
DR	1172600	0.0594 (0.0036)	1.5478	1.7224	1.2277 (0.0161)	0.3153 (0.0222)

GWAS: Genome-Wide Association Studies; SC: Senile cataract; Gla: Glaucoma; AMD: Age-related macular degeneration; DR: Diabetic retinopathy.

Genomic-SEM is robust against biases induced by sample overlap or imbalanced sample sizes. It also facilitates the identification of variants that exert effects on only a subset of complex traits, which consequently do not represent broad cross-trait susceptibility.

Genomic-SEM was conducted in two stages [19]. (1) Estimation of Genetic Covariance Matrices: We prepared summary statistics from GWAS of four AREDs (SC, AMD, Gla, DR) for this stage. Using a multivariate extension of cross-trait LDSC, we derived the empirical genetic covariance matrix among these traits, which served as input for the common-factor SEM model. (2) Model Specification and Evaluation:A structural equation model was specified to minimize discrepancies between the hypothesized covariance matrix and the empirical matrix calculated in Stage 1. Given our primary objective to identify shared genetic architecture across the four ocular traits, we tested a single-factor model. Model fit was assessed using multiple indices: standardized root mean square residual (SRMR), model χ², Akaike information criterion (AIC), and comparative fit index (CFI).

By implementing this common-factor SEM framework, we incorporated individual autosomal SNP associations into the genetic and sample covariance matrices. This yielded a multivariate genome-wide analysis encompassing 4,070,957 SNPs, revealing shared covariance patterns across the GWAS datasets.

### Genomic-SEM SNP heterogeneity

To ensure that the newly identified genomic loci genuinely reflect the shared architecture of the latent ARED factor rather than trait-specific comorbidities, we rigorously applied the Q_SNP_ heterogeneity test. Variants exhibiting significant trait-specific deviations (*P* < 0.05) were systematically excluded. During this filtering process, a total of 412192 variants demonstrating significant heterogeneity were removed. Consequently, all genome-wide significant SNPs reported in this study are strictly interpreted as pleiotropic regulators governing shared age-related ocular vulnerabilities.

### Define genomic loci and identify new variations

We employed FUMA GWAS (Functional Mapping and Annotation of Genome-Wide Association Studies) to identify genomic loci and ascertain lead SNPs associated with the constructed GWAS. These lead SNPs exhibit low linkage disequilibrium (LD) with other SNPs (r^2^ < 0.1) while achieving genome-wide significance (*P*-value < 5 × 10^−8^) [20,21]. We first input summary statistics of SNPs from the constructed GWAS to evaluate their association strengths. Then we compared the lead variant loci against the original univariate GWAS. To assess potential pleiotropic associations of the 12 lead SNPs identified in our new GWAS, we cross-referenced published significant associations (*P*-value < 5 × 10^−8^) in the GWAS Catalog. Functionally, we performed genomic risk locus analysis using FUMA software under a significance threshold (*P*-value < 5 × 10^−8^) and subsequently conducted gene-level association testing via MAGMA (Multi-marker Analysis of GenoMic Annotation). MAGMA—a post-GWAS annotation tool—evaluates gene-trait associations by aggregating genetic markers (e.g., SNPs) into gene-based signals and computing the association of each gene with the phenotype (e.g., disease or health trait), thereby extracting gene-relevant genetic signals from genome-wide SNP data. Significance for gene-based associations was defined as FDR-adjusted *P*-value < 0.05. Additionally, to efficiently discover novel pleiotropic loci with enhanced translational value, we systematically compared the lead variants identified through our Genomic-SEM analysis against those attaining genome-wide significance in the original univariate GWAS.

### Fine mapping

To identify the most probable causal variants associated with ARED, we employed both SuSiE and FINEMAP [22], tools designed for fine-mapping analysis to prioritize putative causal variants linked to a phenotype, implemented within the R package echolocatoR v.2.0.3. The methodological workflow is detailed as follows: A 250-kb genomic window centered on each lead SNP was defined to capture linked variants. For every variant within these regions, the posterior probability of causality (PP) was computed using both tools.Variants surpassing a posterior probability threshold of 0.95 were designated as potential causal variants. This threshold corresponds to a 95% credible set, ensuring high confidence in variant selection. EcholocatoR was utilized to define ‘consensus SNPs’ as variants jointly prioritized by both SuSiE and FINEMAP outputs. For these overlapping variants:The mean posterior probability (mean PP) across tools was calculated. A binary consensus credibility metric (value = 1 if PP ≥ 0.95 in both tools; otherwise 0) was assigned to each variant.

### Whole-transcriptome association study

Following the identification of putative causal variants, we performed a Transcriptome-Wide Association Study (TWAS) to prioritize genes associated with ARED based on inferred relationships between gene expression and the phenotype [23]. This analysis employed the FUSION framework, utilizing precomputed expression quantitative trait loci (eQTL) weights for 37,920 gene-tissue pairs derived from the GTEx v.8 dataset to compute expression-phenotype associations across diverse genes and tissues. For further analysis of TWAS results: The GWAS data for ARED contained sufficient variants to analyze 36,149 gene-tissue features (from an initial set of 37,920 eQTL features), indicating high data quality. Genes meeting the significance threshold (Bonferroni-corrected *P* < 0.05) in their association with the constructed structural equation model were advanced for subsequent analysis. For these TWAS-significant genes, we performed probabilistic fine-mapping using FOCUS (a method specifically designed for TWAS studies).Through FOCUS, we prioritized candidate causal genes in the new GWAS. This approach evaluates gene-phenotype causality by calculating posterior inclusion probabilities (PIP).Integrating prior evidence, we focused on TWAS-significant genes that demonstrated consistency with complementary support (e.g., FOCUS PIP), suggesting their likely causal involvement.

### Gene set and disease ontology enrichment analysis

We conducted gene set enrichment analysis (GSEA) and pathway analysis using MAGMA and FUMA data to investigate potential relationships between ARED and genes implicated in Mendelian disorders and their associated biological pathways. Additionally, we performed enrichment analyses through MendelVar (https://mendelvar.mrcieu.ac.uk/submit/) [24], a specialized platform for Mendelian disease gene annotation.

### Cell annotation analysis

To identify etiological cell types associated with ARED, we employed Cellular Expression Specificity Integration for Complex Traits (CELLECT) [25] with single-cell RNA sequencing data from the Tabula Muris dataset [26]. This resource comprises transcriptomic profiles of 100,000 cells across 20 organs and tissues from Mus musculus. Subsequently, Tabula Muris scRNA-seq data were preprocessed and normalized using CELLEX to compute expression specificity probability scores for each gene. Finally, enrichment analysis for cell-type specificity was performed via LDSC, with cell-type classifications assessed under a false discovery rate (FDR) threshold of 0.05.

### Partitioned heritability estimation via LDSC

To partition the heritability of ARED across various functional genomic categories, we utilized the standard baseline model (version 1.1) within the LDSC framework [27]. This model encompasses 53 overlapping functional categories, including evolutionary conserved regions, coding regions, UTRs, promoters, and intronic regions. Furthermore, it incorporates extensive regulatory and epigenomic annotations (such as DNase I hypersensitivity sites and specific histone marks) sourced from major databases, notably the ENCODE project and the Roadmap Epigenomics Consortium. This comprehensive baseline model was selected as it provides a robust and field-standardized framework to accurately evaluate the enrichment of heritability within specific functional and regulatory elements.

## Results

### Construction of statistical indicators for SEM

Based on LDSC analysis, the heritability contributions (Z-values) for four univariate GWAS inputs were quantified as follows: SC (Z = 12.7), AMD (Z = 3.2), Gla(Z = 8.2), DR (Z = 16.6).The pairwise genetic covariance Z-values were: SC and Gla (Z = 2.94), AMD and Gla(Z = 2.21),SC and AMD (Z = 2.97). (Single-factor genetic parameters are detailed in S2 Table) During SEM preparation, we evaluated the genetic covariance matrix of the four input GWAS datasets against the empirical covariance matrix. The common factor model demonstrated excellent fit:Comparative Fit Index (CFI) = 0.944, Standardized Root Mean Square Residual (SRMR) = 0.030. (Model stability assessmentare are detailed in S3 Table. Latent factors (F1) and univariate structural equation model parameters are detailed in S4 Table) This indicates robust evidence for a shared latent genetic factor, though an evaluation of the standardized loadings reveals that this shared architecture is predominantly driven by the genetic liability of SC. By extending SEM to incorporate individual genetic variations, we generated an indirectly measured GWAS estimating associations between 4,070,957 SNPs and ARED.

### The Genomic-SEM is based on LDSC for genomic control assessment

Through methodological parameter controls, 3199045 SNPs were removed, while 1217311 valid SNPs were retained after regression coefficient filtering. Key genetic statistics are summarized as follows: Mean Chi^2^ = 0.626, Genomic control LambdaGC = 0.938 Maximum Chi^2^statistic = 274.467, Genome-wide significance threshold = 36, Total observed-scale heritability (h^2^) = 0.001 (SE = 0.0001), Intercept term in regression model = 0.5892 (SE = 0.0032). Collectively, multiple estimators confirm that the latent inflation in our structural equation model arises from polygenic heritability signals, rather than population stratification bias or pleiotropic parameter effects. Quality control metrics of the Genomic-SEM GWAS yielded a Mean Chi^2^ of 0.626 and a λGC of 0.938. In the context of multivariable latent factor modeling, these values indicate statistical deflation, which arises from the strict penalization of standard errors required to correct for complex cross-cohort sample overlap. Consequently, this imposes a highly conservative baseline that stringently protects against Type I errors and population stratification. The robust identification of genome-wide significant loci against this conservative background underscores the exceptional strength of the genetic signals, as further visualized by the sharp upward deviation at the extreme right tail of the QQ plot (S1 Fig).

### Evaluation of the structural equation model for ARED based on the FUMA software

**Fig 2:**
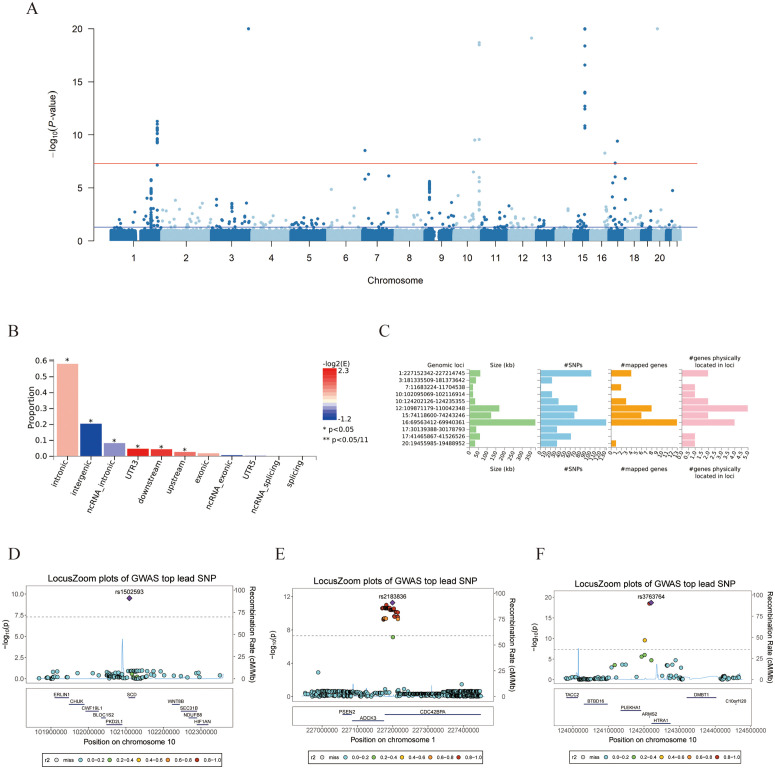
Genomic risk loci identification and annotation for the latent ARED factor using FUMA. **A** Manhattan plot of GWAS summary statistics (only SNPs with *P*-value ≤ 1 × 10^−5^ are kept) **B** Functional consequences of SNPs on genes.**C** Summary per genomic risk locus. **D-F** Risky genetic seat locations detected through FUMA. (rs1502593, rs2183836, rs3763764) GWAS: Genome-Wide Association Study; SNPs: single nucleotide polymorphisms.

Utilizing FUMA software for Genomic-SEM evaluation (Fig 2A-2C), we identified 11 risk gene loci (Fig 2D-2F, S5a Table). Through genome-wide significance thresholds (*P* < 5 × 10^−8^, FDR < 0.05), 7 potential ARED susceptibility genes were prioritized (S5b Table). Furthermore, cross-referencing our identified genomic risk loci with the GWAS Catalog confirms that several of these core genetic regions have been previously implicated in individual age-related ocular conditions (S5c Table). This alignment with established literature robustly validates the biological relevance of the latent ARED construct extracted by our multivariable model. FUMA annotation further revealed 12 lead SNPs, predominantly localized within intronic regions (S6 Table). A total of 12 GWAS subtraction sites were identified (rs2183836, rs1502593, rs36039219, etc.) (S7 Table). rs36039219 is a risk locus for primary open-angle glaucoma reported by Gharahkhani et al. in a multi-ethnic study [28], which is associated with the phenotypic characteristics of our study.

### Fine mapping

**Fig 3:**
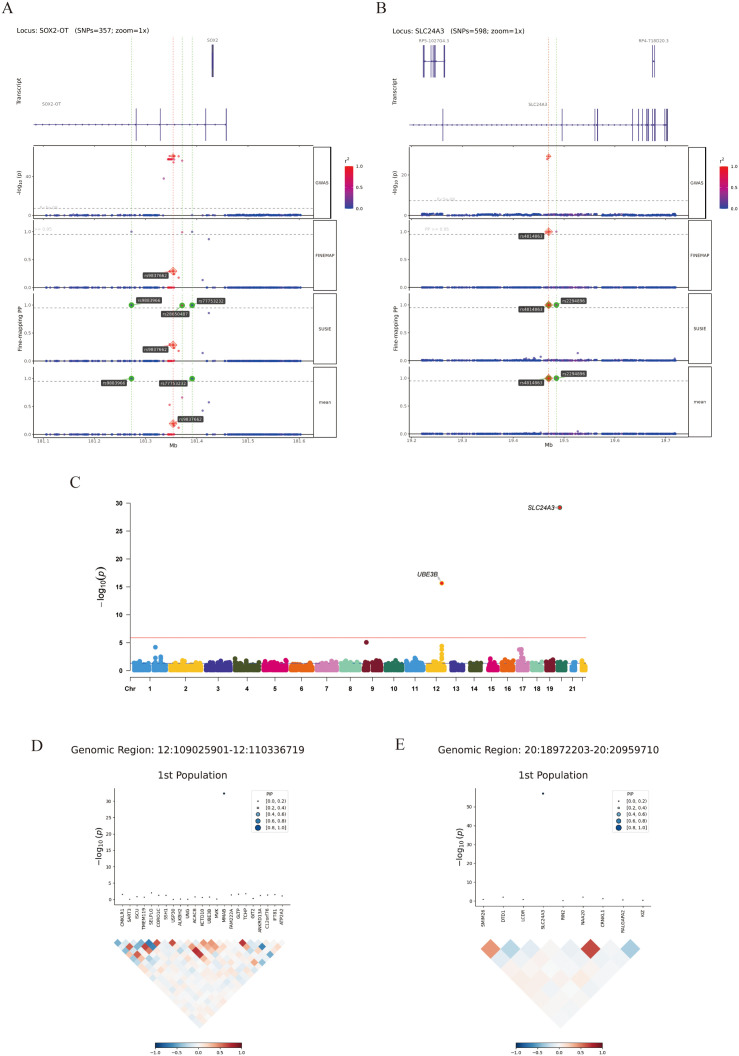
The results of fine mapping and transcriptome prediction. **A,B** Fine localization analysis identified strong associations at multiple genomic locations (mean.PP > 0.95). (SOX2-OT, SLC24A3) **C** Manhattan plot of 2 genes that exceeded the criteria for correction for multiple comparisons from the TWAS. **D,E** FOCUS fine positioning analysis results. (MMAB, SLC24A3) TWAS: Transcriptome-Wide Association Study.

To resolve the broad genomic risk loci identified by FUMA down to the most probable causal variants, we subsequently performed statistical fine-mapping. Fine-mapping analyses revealed robust associations at multiple genomic loci, including: chromosome 3 (rs77753232 and rs9883966, variants in SOX2-OT); chromosome 20 (rs2294896 and rs4814863, variants in SLC24A3); chromosome 7 (rs36039219, a variant in THSD7A); chromosome 10 (rs1502593, a variant in SCD); chromosome 16 (rs11642008, a variant in WWP2) and chromosome 17 (rs35369985, a variant in ARL4D, rs8068039, a variant in CTC-542B22.1). Regional association plots demonstrated distinct peak signals at these loci, with additional credible set variants exhibiting evidence of association (Figs 3A-3B, S8 Table).

### Transcriptome prediction

**Fig 4:**
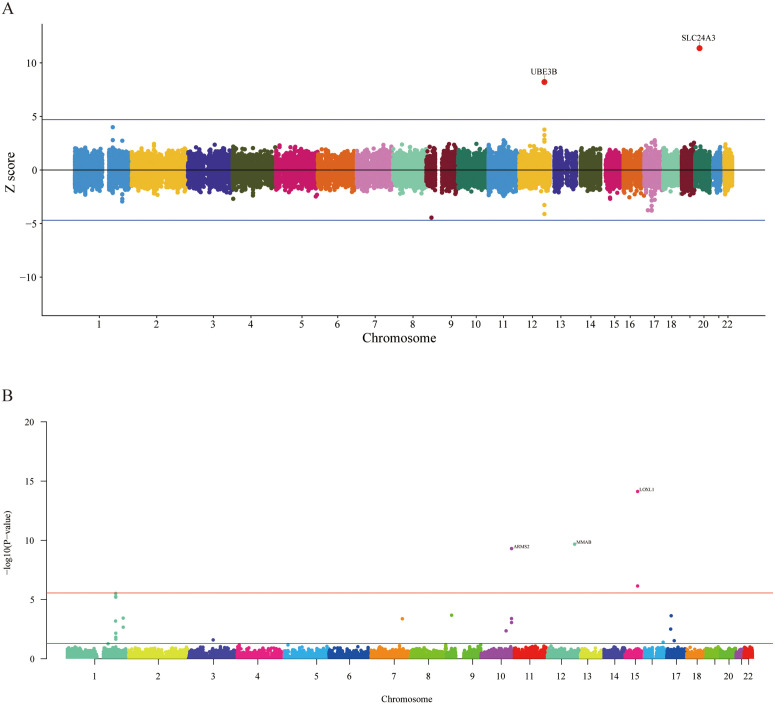
Transcriptomic and gene-based association analyses. **A** Miami plot of Z-scores from the TWAS. **B** Manhattan plot of the gene-based test as computed by MAGMA based on GWAS summary statistics. Genome wide significance (red dashed line in the plot) was defined at *P* = 0.05/17766 = 2.814 *×* 10^−6^; TWAS: Transcriptome-Wide Association Study; MAGMA: Multi-marker Analysis of GenoMic Annotation; GWAS: Genome-Wide Association Study.

Building upon the prioritization of putative causal variants, we next sought to determine how these localized genetic signals translate into functional changes at the gene expression level via a TWAS. We performed a TWAS using FUSION to identify gene-level associations with genetic signatures of ARED. Two genes (SLC24A3 and UBE3B) surpassed the threshold for Bonferroni multiple testing correction (Fig 3C, S9 Table). Subsequent FOCUS fine-mapping analysis applied to Genomic-SEM data revealed 5 genes exhibiting potential causal signals for ARED (S10 Table). To further validate these high-confidence gene-level associations, intersection testing was performed on five prioritized genes: MMAB, SLC24A3, LOXL1,COPRS, and PLEKHA1 (Figs 3D-3E). Following strict multiple-testing correction in the TWAS analysis, the significantly associated genes (such as UBE3B and SLC24A3, shown in Fig 4A, S11 Table) exhibited positive Z-scores (Z > 0), indicating that their genetically predicted upregulation is linked to increased risk for the latent ARED phenotype. While the conservative nature of our multivariable model resulted in a sparse number of transcriptome-wide significant hits, the genes that surpassed the stringent multiple-testing threshold represent highly robust signals.

### Pathways, cell types and enrichment of Mendelian genetic disease genes

**Fig 5:**
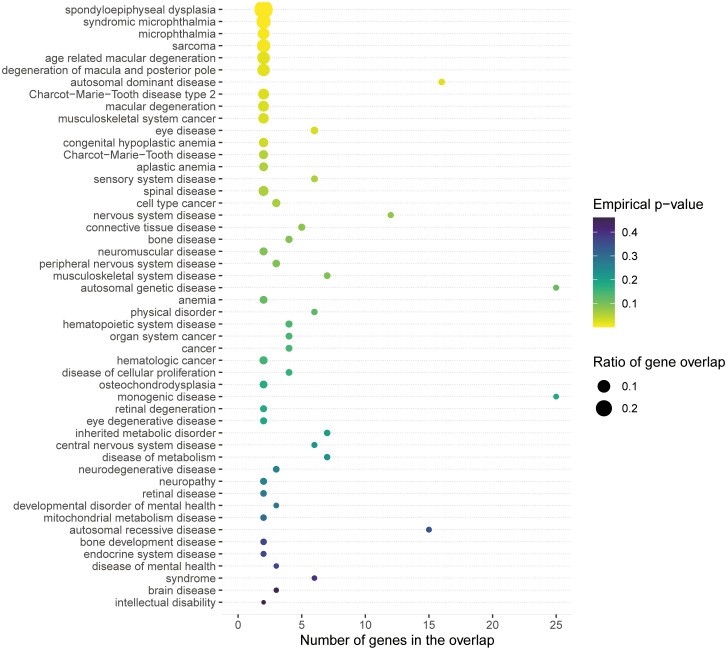
Disease ontology enrichment analysis using MendelVar. The bubble plot illustrates the significant enrichment of mapped genes in specific Mendelian disease categories. The x-axis represents the number of genes overlapping with the specific disease ontology, and the y-axis lists the enriched disease terms. The color gradient of the bubbles reflects the empirical P-value, while the size of the bubbles corresponds to the ratio of gene overlap.

To contextualize these statistically prioritized variants and genes within broader biological networks, we subsequently conducted comprehensive pathway, cell-type, and disease ontology enrichment analyses. MAGMA gene mapping identified four genes (ARMS2, MMAB, TBC1D21, and LOXL1) (Fig 4B, S12 Table). Utilizing these genes for GSEA, we observed significant enrichment across multiple GSEA terms (S13 Table). Some of these gene sets were associated with exfoliative glaucoma and exfoliation syndrome (XFS) [29]. Furthermore, biological processes mapped through MendelVar enrichment were corroborated by GSEA terms, such as spondyloepiphyseal dysplasia (Fig 5). In cell-type-specific enrichment analyses, no significant associations survived FDR correction (S14 Table).

### Heritability contribution at genomic regions

Analysis of heritability contributions across genomic regions revealed that the majority of heritable loci were concentrated in regulatory elements and regions that contain active histone modification marks such as H3K27ac. These regions typically represent critical hubs for gene expression regulation and chromatin remodeling. Notably, histone-modified regions and enhancer elements demonstrated the most significant genetic effects, suggesting that variants in these functional domains may influence trait variability or disease susceptibility through transcriptional modulation (S15 Table).

## Discussion

This study comprehensively investigated the genetic architecture of ARED – including SC, AMD, Gla, and DR,through integrative genomic approaches. By leveraging joint analyses of these complex traits via Genomic-SEM, statistical fine-mapping, and transcriptome-wide analyses, we identified multiple novel genetic markers. Our findings suggest that hereditary factors not only determine susceptibility to ARED but also exert profound lifelong implications through gene-risk factor-cell interplays. This work yields novel theoretical frameworks for understanding how genetic loci shape ocular aging pathologies, informs risk prediction paradigms, and reveals potential targets for non-surgical interventions. Crucially, the latent ARED construct identified in our Genomic-SEM model should be interpreted with caution. Rather than representing a perfectly balanced or singular ‘ocular aging’ biological pathway, this factor likely reflects a highly pleiotropic and composite genetic vulnerability that is primarily anchored by SC-related ocular senescence. This encompasses not only local tissue senescence and shared susceptibility to oxidative and metabolic stressors, but may also capture systemic aging trajectories or shared lifestyle and healthcare utilization effects.

Our study, through Genomic-SEM analysis, revealed significant genetic covariance among SC, AMD, Gla, and DR. The results demonstrate that these phenotypes share common genetic factors, with the LOXL1-AS1 long non-coding RNA (lncRNA) emerging as a highly statistically prioritized candidate region. This aligns with the theoretical framework proposed by Guan et al. (2024), wherein LOXL1-AS1 expression is altered by oxidative stress and cyclic mechanical stress, and its dysregulated expression profoundly impacts global gene expression in pseudoexfoliation glaucoma (PXG) ocular cells [30].Aging represents the predominant risk factor for human chronic diseases, including numerous ocular pathologies [7].These conditions can lead to severe visual impairment and blindness, substantially compromising quality of life [31]. Oxidative stress serves as a key driver in the pathogenesis of ARED such as AMD and DR [32]. With advancing age, critical antioxidants—including glutathione and ascorbate [33]—become depleted, predisposing the eye to increased vulnerability to diverse pathologies [34]. This mechanistic cascade directly contributes to SC pathogenesis through lens protein denaturation and aggregation, while simultaneously acting as a primary instigator of blinding retinal disorders including AMD, Gla, and DR [35]. SEM further corroborated the complex genetic interconnections among SC, AMD, Gla, and DR. These findings indicate that these traits do not exist in isolation but rather function as interwoven biological networks with synergistic effects on disease susceptibility and progression.

Subsequent analyses through Genomic-SEM identified multiple SNPs demonstrating significant associations with ocular pathologies. The majority of these SNPs localize to intronic regions, underscoring the critical regulatory role of introns in genetic mechanisms. Empirical evidence indicates intronic variants modulate gene expression through: RNA splicing alterations, generating protein isoform diversity and functional variability [36]. Pathogenic mutations can occur deep within the introns of more than 75 disease-related genes. Deep intron mutations can disrupt transcriptional regulatory motifs and non-coding RNA genes [37]. These SNP studies related to ARED provide potential genetic targets for subsequent research and offer a new perspective for understanding the genetic connections among ARED.

Through stratified analysis and fine-mapping, this study identified multiple critical SNPs localized within genomic regions associated with macular diseases, glaucoma, and other ocular pathologies. These findings align with Gao et al., who reported 139 genome-wide significant loci linked to macular thickness [38]. The statistical prioritization of these SNPs provides genomic evidence linking these loci to age-related ocular vulnerabilities by modulating macular thickness and retinal disease pathogenesis. Notably, genetic markers in several regions implicated in ocular homeostasis and neurodevelopment suggest these loci play critical roles in shaping ARED. FUSION transcriptome-wide analysis further revealed putative causal genes functionally connected to these SNPs. These genes are predominantly enriched in lipid metabolism, immune response pathways, and other essential biological processes—demonstrating established associations with glaucoma, cardiovascular diseases, and related pathological mechanisms [39,40]. These pathways may play a substantial role in the genetic basis of ARED phenotypes.

Through analysis of whole-genome data, we identified multiple risk-associated chromosomal regions linked to ARED. These regions influence diverse biological processes by modulating the expression of proximal genes. Specifically, risk loci on chromosomes 1, 3, 7, 10, 12, 15, 16, 17, and 20 demonstrate significant associations with aging phenotypes and retinal pathologies, with pronounced enrichment in intronic regions. These genomic elements are statistically implicated in age-related ocular vulnerabilities, leading to the hypothesis that intronic splicing errors might trigger aberrant gene expression and subsequent pathological manifestation [41]. In broader contexts, noncoding genetic variants contribute substantially to complex disease susceptibility. Empirical evidence indicates such variants disrupt disease risk by altering chromatin states and gene regulatory networks, thereby rewiring transcriptional programs in pathological conditions [42,43].

Despite providing novel insights into the genetics of ARED, this study has several limitations. First, our GWAS cohorts primarily comprised individuals of European ancestry, limiting the generalizability of our findings to diverse global populations. Second, as an in silico investigation relying entirely on bioinformatics, the lack of functional validation limits definitive causal interpretation; future targeted experimental models are required. Third, our definition of ARED was methodologically restricted to SC, AMD, Gla, and DR due to the rigorous data requirements of Genomic-SEM, omitting broader clinical conditions like dry eye syndrome. Fourth, the Tabula Muris dataset used for cell-type annotation lacks specialized human ocular cell types, likely accounting for the absence of significant cell-type specific enrichments. Fifth, while integrating stringently controlled public GWAS datasets resulted in statistical deflation—reducing overall power—it conversely ensures that our identified pleiotropic loci represent highly robust signals with an exceptionally low false-positive rate. Sixth, the latent common factor is predominantly driven by SC, inherently weighting the shared genetic architecture toward SC-related biological pathways. Seventh, our ARED factor captures pathways fundamental to systemic aging. Without cross-validation against broad aging-related traits (e.g., lifespan), we cannot definitively distinguish purely ocular-specific pathogenic mechanisms from broader systemic aging susceptibilities. Eighth, because our model already utilized the largest available public GWAS consortia, we lacked an independent, non-overlapping external cohort to validate the latent ARED signal or evaluate polygenic risk scores. Furthermore, the strict intersection of variants across four datasets thinned local SNP density, resulting in some significant loci visually lacking typical LD peaks; these isolated signals require caution pending replication in denser whole-genome sequencing datasets. Finally, although genetic factors substantially contribute to disease pathogenesis, future research must comprehensively explore critical gene-environment interactions.

This study advances understanding of the genetic architecture underlying ARED. By integrating Genomic-SEM, fine-mapping, and transcriptome-wide analyses, we discovered novel genetic loci and described their potential roles in regulating gene expression and complex trait associations. These findings not only deepen mechanistic insights but also inform: Precision medicine approaches for risk stratification. Future work will validate these genetic markers and dissect gene-environment interactions affecting global ocular health outcomes, ultimately advancing vision preservation strategies worldwide.

## Supporting information

S1 Fig QQ plot of ARED GWAS. (TIF)

S1 Table Detailed information of univariate GWAS. (XLSX)

S2 Table Single-factor genetic parameters. (XLSX)

S3 Table Model stability assessmentare. (XLSX)

S4 Table Latent factors (F1) and univariate structural equation model parameters. (XLSX)

S5a Table Risk gene loci. (XLSX)

S5b Table Potential ARED susceptibility genes. (XLSX)

S5c Table Cross-referencing genomic risk loci with the GWAS catalog. (XLSX)

S6 Table Lead SNPs predominantly localized within intronic regions. (XLSX)

S7 Table GWAS subtraction sites. (XLSX)

S8 Table Fine-mapping analyses. (XLSX)

S9 Table Genes exceeding the threshold corrected by Bonferroni multiple testing. (XLSX)

S10 Table FOCUS fine-mapping analysis. (XLSX)

S11 Table Significantly associated genes in TWAS analysis. (XLSX)

S12 Table MAGMA gene mapping. (XLSX)

S13 Table GSEA enrichment. (XLSX)

S14 Table MendelVar enrichment. (XLSX)

S15 Table Heritability contribution at genomic regions. (XLSX)
