# Supplementary figures and images for: Multivariate genetic architecture of age-related eye disease
Source: PLoS One. 2026 May 14;21(5):e0349199. doi: 10.1371/journal.pone.0349199 (PMC13175492; doi:10.1371/journal.pone.0349199)

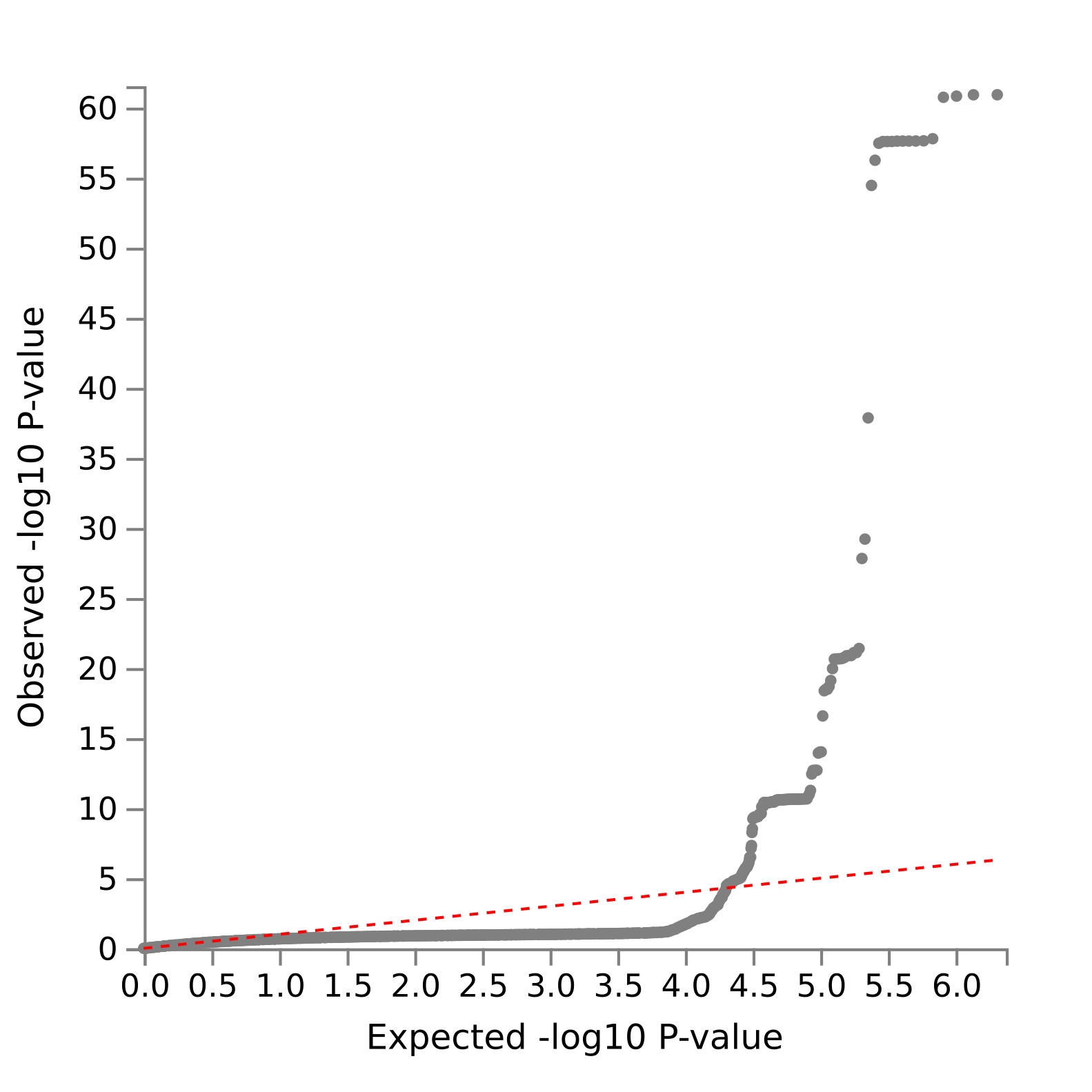

Supplement: S1 Fig — (TIF) [file pone.0349199.s001.tif]
